# The Influence of Exercise Intensity on Tryptophan Metabolites in Thoroughbred Horses

**DOI:** 10.3390/ph16010107

**Published:** 2023-01-11

**Authors:** Magdalena Staniszewska, Sylwester Kowalik, Ilona Sadok, Witold Kędzierski

**Affiliations:** 1 Institute of Health Sciences, Faculty of Medicine, The John Paul II Catholic University of Lublin, Konstantynów 1J, 20-708 Lublin, Poland; 2 Department of Animal Physiology, Faculty of Veterinary Medicine, University of Life Sciences in Lublin, Akademicka 12, 20-033 Lublin, Poland; 3 Department of Chemistry, Institute of Biological Sciences, Faculty of Medicine, The John Paul II Catholic University of Lublin, Konstantynów 1J, 20-708 Lublin, Poland; 4 Department of Biochemistry, Faculty of Veterinary Medicine, University of Life Sciences in Lublin, Akademicka 12, 20-033 Lublin, Poland

**Keywords:** lactic acid, nicotinamide, tryptophan, xanthurenic acid, exercise, Thoroughbred horse

## Abstract

Catabolism of tryptophan (Trp) is modulated by physical activity and provides a pool of active compounds: Trp is considered a calmative agent, kynurenine (Kyn) and 3-hydroxykynurenine (3-HKyn) show neurotoxic effects, kynurenic acid (Kyna) and xanthurenic acid (XA) have neuroprotective properties like nicotinamide (NAm), while serotonin is the neurotransmitter. The study was conducted to investigate the dependence of exercise intensity, measured by plasma lactic acid (LA) concentration, on the level of Trp, its catabolites (serotonin, Kyn, 3-HKyn, Kyna and XA), and NAm in Thoroughbred horses. A total of 18 young race Thoroughbred horses were investigated during exercise tests. Blood samples for analysis were collected: at rest, 10 min after the end of the exercise, and 60 min after the end of the exercise. Plasma LA was determined by the enzymatic method, Trp, and other metabolites using liquid chromatography coupled with mass spectrometry. In horses performing intense exercise, the concentration of LA, Kyn, XA and NAm was increased, while Trp was decreased. Significant correlations were detected for exercise-induced increase in LA and 3-HKyn, XA, and NAm. Considering the scope of changes in analyzed data, there is an expected neutral effect on the health status of exercised horses.

## 1. Introduction

**Figure 1:**
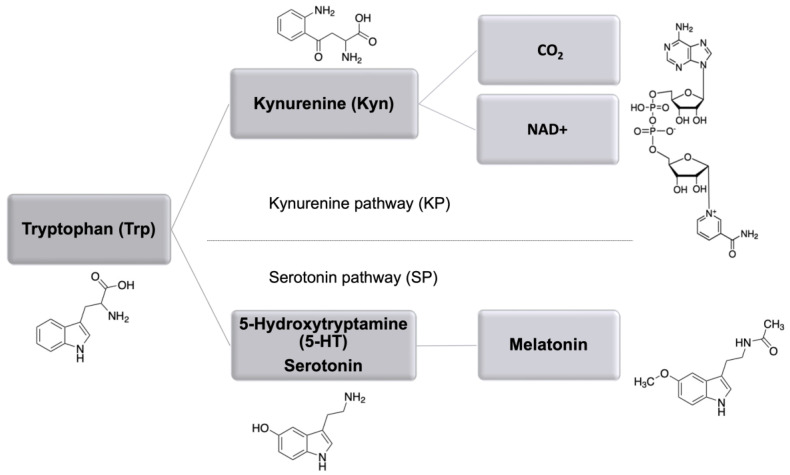
Schematic presentation of the main Trp catabolic routes including kynurenine (KP) and serotonin (SP) pathway with the key metabolites.

In animal and human tissues, tryptophan (Trp) can be used for protein synthesis or degraded following two catabolic routes: serotonin pathway (SP) and kynurenine pathway (KP), leading to the production of melatonin or nicotinamide adenine dinucleotide (NAD+), respectively (Figure 1).

While the majority of kynurenine generated from Trp is completely oxidized to CO_2_ via glutaric acid and acetyl-CoA [1], it also provides a pool of compounds with different biological activities [2]. The SP and KP metabolites play a role in the nervous system as neurotransmitters, like serotonin (5-hydroxytryptamine, 5-HT), or show neurotoxic and depressive effects, i.e., kynurenine (Kyn) and 3-hydroxykynurenine (3-HKyn), while, on the contrary, kynurenic acid (Kyna) and xanthurenic acid (XA) have neuroprotective and anti-depressive properties similarly to NAD precursor nicotinamide (NAm) [3,4,5].

Both mentioned Trp catabolism pathways are modulated by physical activity [6,7]. In athletic horses, significant increases in plasma Trp, 5-HT, Kyn, Kyna and XA in response to different types of exercise were reported [8,9,10,11,12]. The changes in Trp levels were studied also in other horses and showed no important change during trot on the treadmill with increasing speed or cantering lasting 15 min, defined by the authors as medium-heavy exercise [13,14], whereas jumping (the standardized obstacle course) or endurance, low-intense exercise led to a decrease in plasma Trp concentration [12,15]. Additionally, our group has earlier reported that an endurance exercise leads to a decrease in the plasma level of NAm [12]. Thus, probably, the intensity of exercise performed by the horse is the main factor regulating the Trp metabolism. The problem is important considering horses’ health, especially Thoroughbred ones. Thoroughbred horses are primarily bred for racing under saddle at the gallop. They are considered to be the fastest gallopers of all horse breeds. During races, they are encouraged to gallop as fast as they can. Only the best-performing Thoroughbred horses in the races are used for further breeding. Thus, they are routinely submitted to very intense exercise during training and races that test their racing prowess. Therefore, there is a potential for the formation of increased amounts of some neurotoxic and depressive substances (Kyn, 3-HKyn) during repeated exercise bouts in these horses. Other biological effects of the generated KP metabolites are also possible. Namely, an increased Kyn/Trp ratio is associated with inflammation [16], probably associated with increased activity of indoleamine-2,3-dioxygenase, the enzyme included in KP. In addition, Trp is considered a calmative agent; thus, its decrease in response to exercise can negatively impact the horse’s mood and behavior [17]. On the other hand, neuroprotective metabolites (Kyna, XA) can have beneficial effects on the health of the horses experiencing intensive training. Finally, an SP metabolite and biogenic amine 5-HT, in peripheral tissues, regulates gut motility and blood flow due to vasoactive properties and shows immunomodulatory effects [10]. It is widely documented that in horses, circulating 5-HT concentration increases in response to intense exercise [6,11,14].

In practice, the intensity of exercise in galloping horses is evaluated based on blood lactic acid (LA) measurement [18]. LA is produced by the muscle forced to work in anaerobic conditions and then is released into the blood. The amount of LA in the bloodstream is positively correlated with the intensity of exercise and the process of LA release from muscles to the blood is extended in time. Therefore, the blood LA concentration peaks 10 min after the end of exercise in intensively exercised horses [19].

There is not much data on the generation of Trp metabolites during horse training. This study aimed to investigate the influence of exercise intensity (measured by LA concentration in blood) on the plasma level of Trp, its catabolites (serotonin, Kyn, 3-HKyn, Kyna and XA), and NAD precursor (NAm) in Thoroughbred horses.

## 2. Results

We have analyzed the effect of the intensive exercise on the changes in SP and KP selected metabolites in Thoroughbred horses. In the studied group of a high-intense exercise, two horses galloped much slower than the other ones, finishing approximately 30 s later than the others and achieving the plasma LA concentration of 5.22 and 3.87 mmol/L as determined 10 min after the end of a gallop, i.e., many times lower than in the others. The slower run and LA values reaching the level of anaerobic threshold [18] prevented these two horses from fulfilling the task of carrying out high-intense effort (i.e., exercise resulting in an increase in LA to a level of at least 13 mmol/L). The results obtained for these animals have been excluded from further analysis. Thus, the number of horses considered in this group remained at *n* = 16. The determined concentrations of the plasma LA, Trp, and its metabolites quantified in samples from studied horses are shown in Table 1.

Among the studied analytes within the low-intense exercise horses, there was an impact only on the plasma LA concentration showing a slight increase. In contrast, in a group of high-intense exercise, the level of some KP metabolites showed fluctuations with significant changes before and at different time points post-exercise.

**Figure 2:**
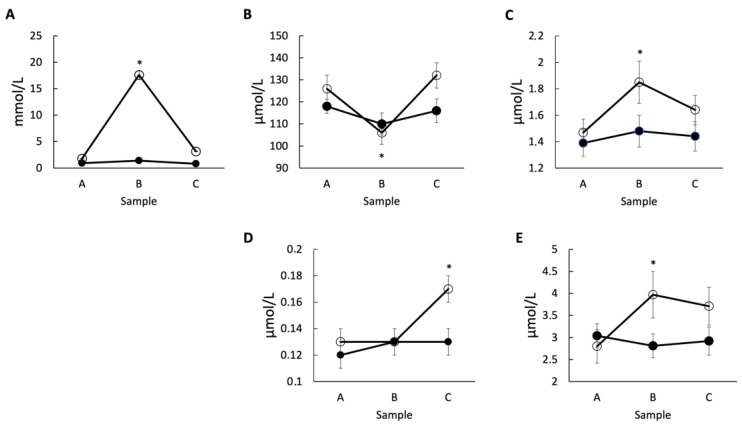
**The metabolites with significant changes related to physical activity.** The concentrations of (**A**) LA, (**B**) Trp, (**C**) Kyn, (**D**) XA, and (**E**) NAm were measured in the samples collected pre-exercise (Sample A), 10 min post-exercise (Sample B), and 60 min post-exercise (Sample C) in the group subjected to the low-intensity (solid circle) or high-intensity (open circle) exercise. Each measurement was performed in triplicates and * indicates *p* < 0.05 relative to sample A.

The concentration of LA, Kyn and NAm in horse plasma was increased post-exercise in the high-intensity exercise (Figure 2A,C,E; open circles) while a decrease in Trp value was observed (Figure 2B). In samples obtained 60 min after the end of intense exercise, the values of LA and XA were significantly higher compared to the resting conditions (Figure 2A,D; open circles). Interestingly, there were no significant changes observed for a level of Kyna, 3-HKyn, and serotonin in the high-intensity exercise group, although the level of serotonin showed a gradual rise post-exercise (Table 1).

The analysis of coefficient correlation was performed for all obtained results (*n* = 36), including those of the two horses which were not taken into account earlier. Values of the coefficient correlation between exercise-induced changes in plasma LA (B-A) and changes in concentrations of the other analytes are presented in Table 2. Statistically significant correlations were determined for post-exercise (B-A) increase in LA and NAm, similar to a correlation for LA (B-A) and the concentration changes measured 60 min after the end of intense exercise and at rest (C-A) for 3-HKyn, XA and NAm. There were no significant changes observed for 5-HT level, regardless of the exercise type or time post-exercise.

## 3. Discussion

In the studied Thoroughbred horses, the high-intense exercise induced a significant decrease in plasma Trp and an increase in Kyn and NAm determined just 10 min after the end of the exercise, as well as a sustained increase in XA detected up to one hour after the end of intense exercise (Table 1, Figure 2). Moreover, the coefficient correlations between the exercise-induced changes in LA and analyzed metabolites indicated that with exercise intensity, there is the increase in the plasma concentration of 3-HKyn, XA and NAm. These results clearly indicate that high-intense exercise influences the kynurenine pathway in Thoroughbred horses. The question is whether these changes may have a significant impact on horses’ health. The noted decrease in plasma Trp concentration was temporary and this effect disappeared after 60 min post-exercise. The return of Trp concentration to the level from pre-exercise was stabilized during the recovery period after racing. It might be associated with a decrease in the activity of the adrenergic nervous system after the end of the exercise, restoration of the vagal system activity, and protein digestion in the gastrointestinal tract. As result, there is a return to the absorption of Trp into the blood that is likely obstructed during high-intensity exercise. This means that a transient drop in Trp is unlikely to have any negative consequences, especially since the NAm concentration in blood increases and potentially facilitates substrate for NAD^+^ synthesis entering the NAD^+^ salvage pathway synthesis [2]. The exercise-induced decrease in Trp was also reported in purebred Arabian horses in response to an endurance effort without a detectable influence on horse mood and behavior [12,20]. The stated increase in Kyn was also transient and did not correlate with the increase in LA. Similarly, the increase in plasma Kyn concentration was noted 30 min after short-time intense exercise in purebred Arabian horses [12]. Kyn is a potentially depression-inducing agent; however, doses ten time higher than physiological levels are known to induce depressive-like behavior in experimental animals [21]. Thus, once again, we believe that the transient increase in Kyn of only 26% resulting from the high-intense exercise reported in the current study is unlikely to cause mental problems. Plasma 3-HKyn concentration did not show significantly important differences in response to exercise such as in Arabian horses studied previously [12]. However, an insignificant increase in 3-HKyn levels observed 60 min after the end of the intense exercise correlated positively with the exercise-induced increase in LA values. This suggests that intense exercise results in increased activity of kynurenine 3-monooxygenase (KMO) catalyzing the conversion of Kyn to 3-HKyn [22]. In the body, 3-HKyn is utilized for the production of XA or QA, leading to NAD^+^ generation. An important source of NAD^+^ supplied with food is niacin and NAm, entering the kynurenine pathway after conversion to nicotinic acid [2]. We observed that plasma concentrations of XA and NAm significantly increased in response to the intense exercise in studied horses, and these changes correlated positively with the increase in LA. Similarly, the increase in XA was reported in intensively exercised purebred Arabian horses [12]. Since XA and NAm show neuroprotective and anti-depressive properties, their increased level in response to intense exercise may counteract the negative influence of the reported change in depressive metabolites. This includes the decrease in Trp and increase in Kyn and 3-HKyn concentrations that is likely further converted into XA observed in our study (Table 1, Figure 2). In the future, it will be interesting to study the changes in the relevant enzyme, mainly increase in activity of kynurenine aminotransferase (KAT) that is responsible for this effect. The animals that are not able to increase KAT activity might be less resistant to the negative impacts of Trp decrease and Kyn, 3-HKyn increase.

In the present study, the exercise-induced changes in circulating 5-HT were statistically insignificant, contrary to other reports [6,10,11,14]. The discrepancy is probably related to the narrow window of 5-HT accumulation in the blood that peaks 30 min after the end of the exercise, as was determined in the cited studies. However, the change may not be as evident as quickly as 10 min after exercise nor later on, e.g., 60 min after the end of the exercise, as was in our case. The lack of significant change in the 5-HT level also suggests no impact on the mood of the studied horses. Serotonin in the body is generated from Trp and the research on dietary supplementation with Trp on horse behavior was conducted by Noble et al. [17]. It showed that despite the supplementation-induced increased Trp level, no evidence of modified behavior was detected in the studied horses.

To summarise, we observed the high-intense exercise-induced increase in Kyn and XA in studied Thoroughbred horses, which agrees with our previously reported studies on the short-time exercise of purebred Arabian horses [12]. Importantly, the high-intense exercise also caused changes in Trp, NAm and 3-HKyn that were not observed earlier. The increase in plasma 3-HKyn, XA, and NAm concentrations resulting from the highly intense exercise correlated with the relative intensity of exercise expressed by the increase in LA levels. The reported changes in the studied metabolites have the potential to influence the horse’s health. A transient decrease in Trp level might limit the production of important, biologically active metabolites, 5-HT and NAD^+^, however, no evidence of that effect was observed. On the contrary, the level of 5-HT was not lowered during the study, whereas plasma concentration of NAm, a metabolite directly used for NAD^+^ synthesis, increased in response to intense exercise. The increased production of Kyn and 3-HKyn might have a negative impact because of their neurotoxic effect, especially Kyn, which can cross the blood–brain barrier and enter the brain. Following oxidation, the generated 3-HKyn has a damaging effect on neurons [23]. Nevertheless, the reported increase in plasma Kyn concentration was relatively low and of short duration. Additionally, the reported increased activity of KAT in exercised muscles results in Kyn transformation to the anti-depressant kynurenic acid and prevents the accumulation of Kyn in peripheral tissues. Thus, Kyn penetration into the brain is inhibited, preventing subsequent depression-inducing consequences [7]. On the other hand, an increase in XA and NAm levels reported in the present study might have neuroprotective and anti-depressive effects. XA also presents antioxidant and vasorelaxation properties in peripheral tissues, in much higher doses than reported in our studies, however [24]. Thus, this effect of the generated XA is unlikely in the analyzed here Thoroughbred horses. Together, considering the scope and type of changes in concentrations of Trp and its metabolites, there is an expected neutral effect on the health status of exercised horses. Intense exercise influenced mainly changes in Trp via neurotoxic Kyn to neuroprotective XA. Since the study considered sampling only at the 60 min post-exercise, it is not clear whether the level of metabolites, especially XA, remains increased or rather decreases during longer post-exercise rest. It will be interesting to study correlation of XA level in time with the animal behavior.

We can only speculate, at the low activity such as at the rest in the physiological state, that the Trp metabolism will return to the initial rate with the basal activity of the KP enzymes and Trp metabolites. In contrast, some pathological conditions such as inflammation might impact enzymatic activity of the KP enzymes, i.e., indoleamine-2,3-dioxygenase and influence the level of Trp metabolites to favor the neurotoxic factors.

## 4. Materials and Methods

### 4.1. Horses

The experiment was carried out with the approval of the Local Ethics Review Committee for Animal Experiments in Lublin (reference number: 45/2017, approval date: 22 May 2017) and was conducted according to European Community regulations. A total of 18 young race Thoroughbred horses were investigated during simulated races, and then the same horses were investigated during routine training sessions. The animals included two-year-old (5 stallions and 3 mares), three-year-old (5 stallions and 2 mares), and four-year-old horses (3 stallions). All horses were trained by a single professional trainer on a racetrack. The stallions and mares were kept in two separate stables in 4 m × 4 m boxes. They were fed and taken care of in the same manner used for racehorses. Each horse received an individually calculated ration of oats, hay, and feed concentrate. The food ration reflects the horse’s needs depending on workload and body condition, including an average digestible energy of 0.26 MJ/kg body weight (BW) per day and digestible protein of 2.0 g/kg BW/day, distributed over three feeds per day. Fresh water and mineral salt block were available without restrictions. The training was started three months before the experiment. Before the study, the horses were exercised five times a week. The routine training session consisted of 15 min of walking and 10 min of trot or canter as a warm-up, and then gallop over 600–1200 m distance, at a speed of 6–14 m/s, depending on the horse’s performance, and finally 10 min of trot and walk. After the exercise, each horse was cooled down on an automatic horse walker for 45 min. The study was performed in May, which coincided with the beginning of the race season. The samples for Trp metabolites and LA were taken from all 18 horses twice to investigate (1) the intense training session of galloping in groups of four or five (simulated races) and (2) the low-intensive training session of canter after warm-up only, without gallops one-week post-intense training session. Taking into account the young age of the horses under study, it was assumed in this study that intense exercise should increase LA concentration in the blood to a value of at least 13 mmol/L [25]. All studied horses were inspected by a veterinarian and behaviorist for the presence of musculoskeletal or behavioral disturbances. None of the horses tested showed any signs of health or mental disturbances.

### 4.2. Blood Samples Collection and Analysis

On the day of the study, three blood samples were collected from each horse: at rest, in the stable (A), then 10 min after the end of gallop or canter (B), and finally 60 min after the end of gallop or canter, immediately after the end of cooling down on an automatic horse walker (C). Blood samples were taken by jugular venepuncture into tubs containing sodium–potassium EDTA, immediately cooled down by being put into a water bath at a temperature of 4 °C and then centrifuged at 2000× *g* for 10 min for plasma separation. The plasma LA concentration was measured immediately by the use of a commercial kit (Liquick Cor-LACTATE, Cormay, Warsaw, Poland) and a portable analyzer (Dr Lange Laboratory System LP450, Germany). The remaining plasma was frozen and stored at −80 °C until further analysis.

Trp, NAm, 5-HT and kynurenine pathway metabolite levels (Kyn, Kyna, 3-HKyn, XA) were determined using the Ultra-High Performance Liquid Chromatography-Electrospray Ionization-Tandem Mass Spectrometry (UHPLC-ESI-MS/MS) in plasma after protein precipitation with trichloroacetic acid, according to our previously published protocol [12,26]. In the current study, the analysis has been extended to an additional metabolite–serotonin (5-HT). The serotonin concentration was calculated based on a standard compound (Sigma-Aldrich, St Louis, MO, USA) by setting the transitions: 177.1 > 160.0 (quantifier, fragmentor: 80 V; collision energy: 10 eV) and 177.1 > 132.0 (qualifier, fragmentor: 80 V; collision energy: 26 eV). Data were acquired using a dynamic multiple reaction monitoring (dMRM) mode with positive polarity. Samples were analyzed on a 1290 infinity UHPLC system connected to a 6460 triple quadrupole mass spectrometer (Agilent Technologies, Santa Clara, CA, USA) equipped with an electrospray ion source (Agilent Jet Stream). Separation of the target compounds was achieved on the Zorbax Eclipse Plus-C18 HPLC column (2.1 mm × 100 mm × 1.8 µm) protected by the Zorbax Eclipse Plus-C18 Guard Column (2.1 mm × 12.5 mm × 1.8 µm) using condition described earlier [12]. Quantitative analysis was performed based on matrix-matched calibration curves prepared in the presence of a fixed amount of 3-nitrotyrosine used as an internal standard (Sigma-Aldrich, St Louis, MO, USA). Each sample was injected in triplicates.

### 4.3. Statistical Analysis

For statistical analysis, the software package GraphPad Prism^TM^ 6, version 6.01 was used (Graph Pad Software, La Jolla, CA, USA). Firstly, data were tested for normality of distribution by the Shapiro–Wilk test. A normal distribution was determined. Thus, data were analyzed using one-way ANOVA for repeated measures (at rest, 10 min after the end of gallop or canter, and 60 min after the end of gallop or canter) separately for high-intense exercise and low-intense exercise. Tukey’s test as the post hoc comparison of analyzed results was used. The results are quantified as means ± SE. The coefficient correlation was also carried out to compare the exercise-induced changes in plasma LA (B-A), and Trp and its metabolites studied (B-A and C-A) using Pearson’s test (*r*). Statistical significance was accepted at the level of *p* ≤ 0.05.

## 5. Conclusions

Different intensity of exercise modulates the Trp metabolism in peripheral tissues, especially impacting KP in Thoroughbred horses. The intense exercise measured by blood LA concentration induced a proportional increase in plasma 3-HKyn, XA and NAm concentration measured in blood samples collected after the training session. More research is needed to explain the influence of increased amounts of these metabolites on horse mood and health. The focused analysis of the KP enzymes involved in the generation of the accumulating metabolites is warranted to explain the effect of an exercise. It will also be interesting to verify whether the supplementation of diet with Trp and NAm that balances the negative effect of the neurotoxic Trp metabolites might have a positive consequence on the post-exercise mood in the intensively trained horses.

## Figures and Tables

**Table 1:** Plasma tryptophan and its metabolites determined in trained racehorses during the exercise of different intensities (means ± SE).

Analyzed Parameters	Low-Intensity Exercise (*n* = 18)			High-Intensity Exercise (*n* = 16)			
	Sample A	Sample B	Sample C	Sample A	Sample B	Sample C	*p*-Value
Lactic acid [mmol/L]	0.92 ± 0.04	1.38 ± 0.08 *	0.81 ± 0.03	0.87 ± 0.04 ^a^	16.2 ± 0.83 ^b^	2.32 ± 0.06 ^c^	0.0007
Other analytes [µmol/L]:							
Tryptophan	118 ± 6.17	110 ± 5.28	116 ± 5.74	126 ± 3.24 ^a^	106 ± 4.98 ^b^	132 ± 5.36 ^a^	0.0010
Kynurenine	1.39 ± 0.10	1.48 ± 0.12	1.44 ± 0.11	1.47 ± 0.10 ^a^	1.85 ± 0.16 ^b^	1.64 ± 0.11 ^ab^	0.0212
Kynurenic acid	0.22 ± 0.03	0.22 ± 0.04	0.22 ± 0.03	0.20 ± 0.03	0.18 ± 0.03	0.19 ± 0.03	>0.05
3-Hydroxykynurenine	0.70 ± 0.04	0.62 ± 0.03	0.65 ± 0.04	0.65 ± 0.07	0.68 ± 0.06	0.74 ± 0.07	>0.05
Xanthurenic acid	0.12 ± 0.01	0.13 ± 0.01	0.13 ± 0.01	0.13 ± 0.01 ^a^	0.13 ± 0.01 ^a^	0.17 ± 0.01 ^b^	0.0007
Nicotinamide	3.04 ± 0.27	2.81 ± 0.27	2.92 ± 0.32	2.80 ± 0.38 ^a^	3.97 ± 0.53 ^b^	3.71 ± 0.43 ^ab^	0.0371
Serotonin	0.21 ± 0.03	0.21 ± 0.03	0.24 ± 0.03	0.18 ± 0.02	0.21 ± 0.02	0.23 ± 0.04	>0.05

A—blood sample taken at rest, B—10 min after the end of intense exercise (gallop or canter), C—60 min after the end of intense exercise; *p*-value is given for the statistical influence of analyzed factor (ANOVA); * indicates *p* = 0.016 as compared to Sample A; ^a^, ^b^, ^c^—mean values in rows marked with different letters differ significantly according to the Tukey’s test.

**Table 2:** Correlations between exercise-induced changes in plasma LA (B-A) and other analytes determined in all horses studied (*n* = 36).

Analytes:	B-A	C-A
LA (B-A) and: Tryptophan	−0.295	0.21
Kynurenine	0.24	0.12
Kynurenic acid	−0.11	0.07
3-Hydroxykynurenine	0.33	0.42 *
Xanthurenic acid	0.02	0.63 *
Nicotinamide	0.56 *	0.61 *
Serotonin	0.19	0.15

*—correlations statistically significant (*p* ≤ 0.05).

## Data Availability

Not applicable.
